# Supplementary material for: Multi‐dataset identification of innovative feature genes and molecular mechanisms in keratoconus
Source: J Cell Mol Med. 2024 Sep 19;28(18):e70079. doi: 10.1111/jcmm.70079 (PMC11412914; doi:10.1111/jcmm.70079)
Supplement: Supplementary file 2 — Material S2. [file JCMM-28-e70079-s002.zip › 7.GSEA analysis/1.ARL11/KEGG_RNA_DEGRADATION.html]

Details for gene set KEGG\_RNA\_DEGRADATION[GSEA]

|  || Dataset | ARL11.group.cls#h\_versus\_l |
| Phenotype | group.cls#h\_versus\_l |
| Upregulated in class | h |
| GeneSet | KEGG\_RNA\_DEGRADATION |
| Enrichment Score (ES) | 0.19066845 |
| Normalized Enrichment Score (NES) | 0.9855557 |
| Nominal p-value | 0.5135659 |
| FDR q-value | 1.0 |
| FWER p-Value | 1.0 |
Table: GSEA Results Summary

  

Fig 1: Enrichment plot: KEGG\_RNA\_DEGRADATION      
 Profile of the Running ES Score & Positions of GeneSet Members on the Rank Ordered List

  

| SYMBOL | TITLE | RANK IN GENE LIST | RANK METRIC SCORE | RUNNING ES | CORE ENRICHMENT || 1 | DIS3 | na | 5714 | 0.145 | -0.0972 | Yes |
| 2 | CNOT1 | na | 7255 | 0.107 | -0.0918 | Yes |
| 3 | ZCCHC7 | na | 7814 | 0.096 | -0.0631 | Yes |
| 4 | CNOT6L | na | 8220 | 0.088 | -0.0337 | Yes |
| 5 | CNOT2 | na | 8898 | 0.076 | -0.0176 | Yes |
| 6 | EXOSC6 | na | 9118 | 0.073 | 0.0103 | Yes |
| 7 | ENO2 | na | 9323 | 0.070 | 0.0370 | Yes |
| 8 | DDX6 | na | 9597 | 0.066 | 0.0599 | Yes |
| 9 | ENO3 | na | 10243 | 0.057 | 0.0679 | Yes |
| 10 | CNOT10 | na | 10660 | 0.052 | 0.0801 | Yes |
| 11 | CNOT6 | na | 10676 | 0.051 | 0.1037 | Yes |
| 12 | DCP1B | na | 12575 | 0.031 | 0.0633 | Yes |
| 13 | PARN | na | 12775 | 0.029 | 0.0711 | Yes |
| 14 | PAPOLA | na | 12883 | 0.028 | 0.0811 | Yes |
| 15 | PAPOLB | na | 13075 | 0.026 | 0.0879 | Yes |
| 16 | DCPS | na | 13091 | 0.026 | 0.0997 | Yes |
| 17 | LSM3 | na | 13318 | 0.024 | 0.1045 | Yes |
| 18 | EXOSC9 | na | 13977 | 0.019 | 0.0944 | Yes |
| 19 | EXOSC3 | na | 14244 | 0.017 | 0.0947 | Yes |
| 20 | EXOSC4 | na | 14773 | 0.013 | 0.0855 | Yes |
| 21 | ENO1 | na | 14982 | 0.011 | 0.0847 | Yes |
| 22 | LSM7 | na | 15215 | 0.010 | 0.0825 | Yes |
| 23 | DCP2 | na | 15396 | 0.008 | 0.0812 | Yes |
| 24 | EXOSC2 | na | 15480 | 0.008 | 0.0824 | Yes |
| 25 | EXOSC1 | na | 15510 | 0.007 | 0.0850 | Yes |
| 26 | CNOT3 | na | 16105 | 0.003 | 0.0693 | Yes |
| 27 | XRN1 | na | 16473 | 0.000 | 0.0589 | Yes |
| 28 | EXOSC8 | na | 17324 | -0.006 | 0.0370 | Yes |
| 29 | LSM1 | na | 17790 | -0.009 | 0.0277 | Yes |
| 30 | DCP1A | na | 18057 | -0.011 | 0.0250 | Yes |
| 31 | EDC4 | na | 18661 | -0.015 | 0.0145 | Yes |
| 32 | HSPD1 | na | 18702 | -0.015 | 0.0203 | Yes |
| 33 | EDC3 | na | 18834 | -0.016 | 0.0240 | Yes |
| 34 | LSM8 | na | 19493 | -0.020 | 0.0145 | Yes |
| 35 | EXOSC10 | na | 19648 | -0.021 | 0.0201 | Yes |
| 36 | LSM5 | na | 20293 | -0.026 | 0.0136 | Yes |
| 37 | PAPOLG | na | 20752 | -0.029 | 0.0142 | Yes |
| 38 | LSM4 | na | 21469 | -0.035 | 0.0098 | Yes |
| 39 | PATL1 | na | 21602 | -0.036 | 0.0228 | Yes |
| 40 | HSPA9 | na | 21794 | -0.038 | 0.0349 | Yes |
| 41 | LSM2 | na | 22090 | -0.041 | 0.0454 | Yes |
| 42 | CNOT7 | na | 22733 | -0.047 | 0.0490 | Yes |
| 43 | XRN2 | na | 22746 | -0.047 | 0.0708 | Yes |
| 44 | PNPT1 | na | 23168 | -0.052 | 0.0830 | Yes |
| 45 | C1D | na | 23228 | -0.053 | 0.1058 | Yes |
| 46 | CNOT4 | na | 23394 | -0.054 | 0.1265 | Yes |
| 47 | EXOSC7 | na | 23436 | -0.055 | 0.1510 | Yes |
| 48 | LSM6 | na | 23466 | -0.055 | 0.1760 | Yes |
| 49 | CNOT8 | na | 25875 | -0.088 | 0.1477 | Yes |
| 50 | EXOSC5 | na | 26359 | -0.096 | 0.1786 | Yes |
| 51 | MPHOSPH6 | na | 28116 | -0.134 | 0.1907 | Yes |
Table: GSEA details [plain text format]

  

Fig 2: KEGG\_RNA\_DEGRADATION      
 Blue-Pink O' Gram in the Space of the Analyzed GeneSet

  

Fig 3: KEGG\_RNA\_DEGRADATION: Random ES distribution      
 Gene set null distribution of ES for **KEGG\_RNA\_DEGRADATION**
